# A community resilience index for place‐based actionable metrics

**DOI:** 10.1111/risa.17684

**Published:** 2024-12-06

**Authors:** Margot Habets, Susan L. Cutter

**Affiliations:** ^1^ Department of Geography University of South Carolina Columbia South Carolina USA

**Keywords:** BRIC, geographic scale, resilience metric, South Carolina

## Abstract

Community resilience measurement to natural hazards is becoming increasingly relevant due to the growth of federal programs and local and state resilience offices in the United States. This study introduces a methodology to co‐produce an actionable resilience metric to measure locally relevant and modifiable indicators of community resilience for the state of South Carolina. The “actionable” metrics, based on the Baseline Resilience Indicators for Communities (BRIC) index, are calculated at the county and tract scale and then compared to “conventional” versions of BRIC. Actionable BRICs perform better in reliability testing than conventional BRICs. Correlations across the two scales of BRIC construction show a stronger relationship between the actionable BRICs than conventional, though all are highly correlated. When mapped, actionable BRIC shows a shifted region of low resilience in the state when compared to conventional BRIC, suggesting that actionable and conventional BRICs are distinct. Scale differences show dissimilar drivers of resilience, with county‐level resilience driven by community, social, and environmental resilience and tract‐level resilience driven by social and institutional resilience. Actionable tract‐level BRIC appears to be the best representation of modifiable resilience for South Carolina, but it comes with trade‐offs, including calculation complexity and changing geographies over time. Regardless of scale, the resulting actionable indices offer a useful tracking mechanism for the state resilience office and highlight the importance of integrating top‐down and bottom‐up resilience perspectives to consider local drivers of resilience. The resulting methodology can be replicated in other states and localities to produce actionable and locally relevant resilience metrics.

## 1 INTRODUCTION

Citing increasing hazard threats and severity, many national and international agencies have prioritized community resilience to lessen hazard impacts and reduce time to recovery (United Nations International Strategy for Disaster Reduction, 2015). In the United States, community resilience measurement is integrated into the Federal Emergency Management Agency's National Risk Index (NRI), and many states have an office or program focused on resilience to natural hazards. In addition, federal legislation now provides direct funding and assistance to specific census tracts called Community Disaster Resilience Zones, identified through a combination of the NRI and the Climate and Economic Justice Screening Tool (Community Disaster Resilience Zones Act P.L. 117–255).

States and municipalities are increasingly creating positions and offices related to community resilience (Morley, 2023) that need usable tools to assist in resilience planning, decision‐making, and tracking progress. However, many offices have yet to adopt existing tools, suggesting a possible misalignment between research‐developed metrics and practical needs (National Academies of Sciences, Engineering, and Medicine [NASEM], 2019). This paper modifies the Baseline Resilience Index for Communities (BRIC) to create a locally relevant and actionable resilience index for South Carolina. Co‐produced with the newly created South Carolina Office of Resilience (SCOR), this index only uses characteristics that can be directly changed at the state or local scale through nonlegislative action so that the index is suitable for monitoring resilience over time, identifying project areas, and allocating resources as they directly relate to resilience outcomes for the state.

By taking a practical perspective on a research‐developed resilience index for this paper, we seek to answer the following questions:

1. How can a state‐level resilience index be designed to measure relevant community characteristics that can be modified by local or state action?
2. How does this index compare to conventional national resilience indices?

For this study, BRIC is customized for the county (CBRIC) and census tract scale (TBRIC) for the state of South Carolina. To accurately reflect resilience priorities and modifiable characteristics relevant to the state, and to ensure that the resulting indices are applicable for practical use, variable selection and aggregation are conducted in partnership with SCOR.

## 2 CURRENT COMMUNITY RESILIENCE MEASUREMENT

The goals of increased resilience include reducing hazard impacts, minimizing the time to recovery after a hazard event, and reducing vulnerability to future hazards (Koliou et al., 2018; Tariq et al., 2021). Resilience measurement aims to provide evidence for allocating funds, implementing policies and practices, and tracking resilience progress over time (National Research Council, 2012; Zuzak et al., 2022). Though many resilience measurement methods exist, there is no dominant framework or standard for resilience measurement because communities differ in physical, social, and built environment characteristics, disaster risk exposures, and capacities (Cutter, 2016).

Beyond identifying the most applicable technique for measuring resilience, which depends on scale, data availability, and the conceptual framing of resilience, measurement schemas must also be usable and useful (NASEM, 2019). Despite the many tools available, few communities explicitly measure their resilience, demonstrating a disconnect between tool development and implementation (NASEM, 2019). For research to be usable for practitioners, it must align with the challenges they encounter, demonstrate credibility, and possess legitimacy, ensuring unbiased findings (Wall et al., 2017). Current resilience measurement tools can be challenging and time‐consuming to use and are not always relevant to sub‐national resilience priorities. Indices may substitute local applicability and specific intended uses for widespread applicability, making it difficult for practitioners to identify relevant and effective actions for their jurisdiction.

In addition, many current resilience indices measure contextual and actionable characteristics under the umbrella of holistic community resilience. Social and place contexts, such as the age of a town's residents or the distance of a place to a state's capital, are relevant for community resilience measurement but are not changeable by direct action. Alternatively, actionable characteristics, such as the number of households with broadband internet or the number of structures in a flood zone, are changeable through nonlegislative actions by state or local agencies. Resilience measures that combine contextual and actionable characteristics help researchers and practitioners understand a place's community resilience but can make it difficult for a state or municipality to pinpoint specific actions to improve it.

There is a need for resilience indices representing a more local perspective while prioritizing actionable metrics. This paper provides a methodology where BRIC is customized and co‐developed with SCOR as a proof of concept—prioritizing local application and actionable metrics over a broader representation of community resilience.

## 3 BRIC

BRIC was developed by the Hazards Vulnerability and Resilience Institute at the University of South Carolina to measure the inherent qualities of communities as they relate to resilience through 50 variables sorted into six distinct capitals (Cutter et al., 2014; Cutter et al., 2010; Hazards Vulnerability and Resilience Institute [HVRI], 2024). These capitals are human well‐being (social), economic/financial capital, community capacity, institutions and governance, infrastructure and housing, and environmental/natural capital, and are commonly used in resilience measurement (Sharifi, 2016). The index is rooted in the Disaster Resilience of Place model, which states that the inherent conditions of a community, including its resilience, vary from place to place and impact how a community or region recovers from a hazard event (Cutter et al., 2008).

BRIC has been critically evaluated (Camacho et al., 2023; McConkey & Larson, 2022) and adapted to new study areas (Javadpoor et al., 2021; Pazhuhan et al., 2023; Singh‐Peterson et al., 2014). In addition, BRIC has been assessed over time (Cutter & Derakhshan, 2020), downscaled for the Gulf Coast region (Derakhshan et al., 2022), and is currently used in the NRI (Zuzak et al., 2023). The ability of BRIC to be adapted to new study areas and different scales makes it an appropriate methodology for this study. Using place‐specific and co‐developed variables and data to calculate a BRIC relevant to and useable in South Carolina is a major contribution to moving science to practice.

The geographies of resilience are examined at both county and census tract scales to further examine what variables are relevant to resilience at different scales. Since the optimal scale of analysis for resilience measurement is unknown, data are aggregated and downscaled to calculate both versions of BRIC presented here, resulting in scaling problems described by the Modifiable Areal Unit Problem (MAUP) (Buzzelli, 2020). In addition, zonal effects are caused by converting data from one set of boundaries to another. The zonal and scale effects of aggregating and downscaling data can have inconsistent and misleading results (Chu et al., 2021; Schuurman et al., 2007); however, these differences can also reflect the different resilience processes operating at the two geographical levels (Wong, 2004).

## 4 DATA AND METHODOLOGY

**FIGURE 1:**
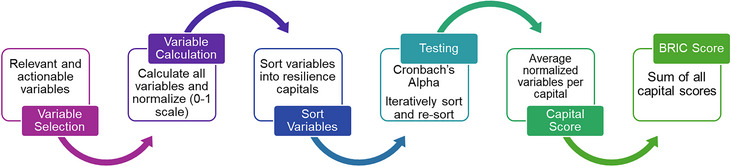
Methodology for construction actionable CBRIC and TBRIC.

To create actionable CBRIC and TBRIC (referred to as CBRIC_A_ and TBRIC_A_) for South Carolina, an initial variable list was created in partnership with SCOR. The methodology to calculate actionable BRICs is depicted in Figure 1 and described below. The actionable CBRIC and TBRIC are then compared to conventional versions of BRIC that have been rescaled to South Carolina.

### 4.1 SCOR partnership and co‐development of variable selection

The South Carolina state legislature founded SCOR in 2020 to serve the state in three ways: (1) create and implement a Statewide Resilience Plan, (2) help the state mitigate against future flood risks, and (3) assist in housing recovery following a federally declared disaster (SC Resilience Revolving Fund Act SC Code §48‐62‐10), making them a suitable partner in this research.^1^ The Statewide Resilience plan was released in 2023 (South Carolina Office of Resilience [SCOR], 2023).

A first meeting with SCOR took place in November 2022 and covered the agency's outlook on community resilience, its vision for the custom indices, and its role in the project. SCOR has defined resilience as, “the ability of communities, economies, and ecosystems within South Carolina to anticipate, absorb, recover, and thrive when presented with environmental change and natural hazards” (SCOR, 2023, p. ii). SCOR's role as the agency partner was essential in advising on the applicability of variables to a South Carolinian context and ensuring that the resulting index was locally relevant and useful for their purposes.

The first stage of creating actionable BRICs was identifying and selecting actionable and relevant variables for South Carolina. A second meeting with SCOR explicitly aimed to review possible variables for the actionable BRICs for the state, starting with a preliminary list of variables from previous BRIC constructions for the United States. Two overarching questions helped guide the discussion to determine how well a variable could represent actionable resilience in South Carolina. First, is the variable relevant to a statewide South Carolina context? Second, is the variable changeable through a direct or indirect action by a state or local agency, whether SCOR or other, using nonlegislative action? The initial list of variables was amended to remove contextual and unactionable variables and those determined as locally irrelevant. In the same process, a wish list of actionable variables was drawn from the literature and SCOR's knowledge of state and local practices. Local data sources that could replace national datasets used in previous BRIC constructions were also identified.

Once candidate variables were reviewed, a revised list of variables was produced using three categories: approved, rejected, and further clarification needed. At the third meeting with SCOR, the variables needing further clarification were discussed and decided upon, and the need to adjust calculations for the census tract geography was discussed (see Section 4.2). A final list of co‐developed variables was produced (Table S1) and sourced from South Carolina datasets when available; otherwise, from national datasets. Datasets were considered for their completeness, legitimacy (e.g., federal agency, research group), usability for both county and census tract scales, and whether they were regularly updated.

### 4.2 Variable calculation

To calculate the data for CBRIC_A_ and TBRIC_A_, spatial data were either aggregated (e.g., point data summarized by geography), converted to different spatial scales (e.g., averaged zip code data), or allocated across geography (e.g., county GDP allocated to all tracts). These calculations used shapefiles from 2019 for county‐ and census‐tract boundaries downloaded from the Census Bureau, with 18 census tracts removed from the analysis as they had no population or no housing units within the tract. This geography was used so any data created in the last 10 years could be utilized in the indices. This is especially important for census tract geography, which changed in 2020 for the new decennial census. Crop insurance was only calculated at the county scale as no tract‐level data were available, and crops vary too much within a county to assign all tracts the same value. Travel time to medical care was only calculated for census tracts as most counties contained a hospital, resulting in a travel time of zero.

Few datasets had null or missing values as point datasets were assumed complete (i.e., no points within a county or tract are recorded as a zero, not a missing or null value), and census data were complete for South Carolina at county and tract geographies. Dam age and bridge sufficiency contained missing values for certain structures, and these were replaced with the state's median value in CBRIC and the county median value in TBRIC before variable calculation. In two other datasets for tract‐level analysis—life expectancy and asthma—a small number of census tracts were missing data and were imputed using the county average where the tract was located. Lastly, Environmental Protection Agency (EPA)‐registered facility data contained duplicate facilities, which were consolidated by registry ID before calculating variables on air quality, water quality, and toxic facilities.

**FIGURE 2:**
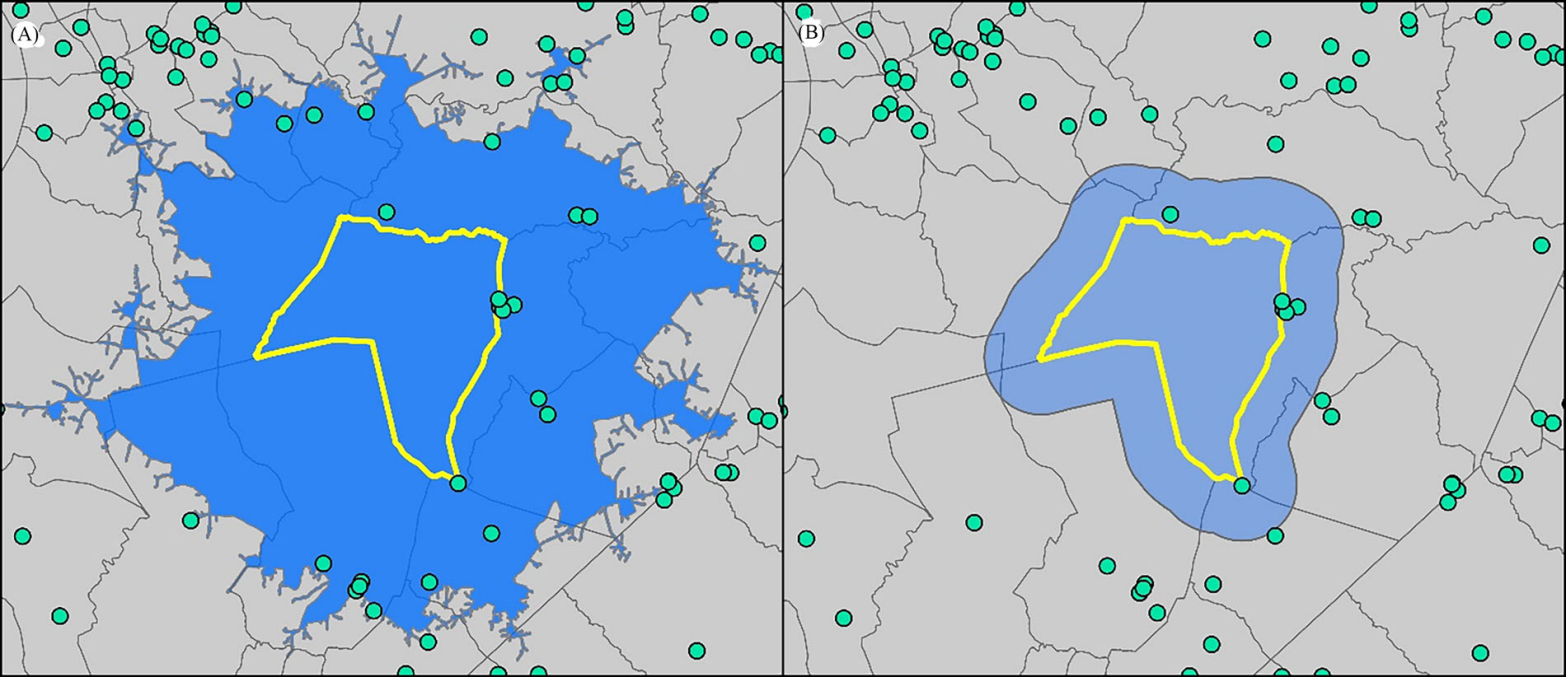
Examples of the effect of the (A) 30‐min access buffer and (B) 5‐km proximity buffer for a census tract and example point data.

In addition to requiring data transformations, census tracts presented an additional representational problem as they do not align with subcounty municipal boundaries and are geographic units used only for statistical purposes. Due to this challenge, several variables are measured by what is only located within the census tract and, alternatively, with an access or proximity buffer to model what is located within and near census tracts. The two types of buffers were determined depending on whether the variable measures a person's access to a specific resource (e.g., transit stops and food pantries) or proximity, where walking or driving time do not matter (e.g., air pollutants and mines) (Figure 2) (Logan & Guikema, 2020).

The access buffer considered urban–rural differences between access expectations and was applied to 11 variables. Census tracts were defined as urban if 30% or more of the tract land area was located within a municipality or census‐designated place (Figure S1).^2^ Updated Rural‐Urban Continuum Codes codes, which are often used to identify urban and rural areas, were not available at the time of calculation, but the method adopted here was confirmed with SCOR to represent urban areas in the state accurately. For urban tracts, access was defined as a 15‐min walk (approximately 2 km) from the centroid of the tract, and in rural tracts, access was defined as a 30‐min drive (approximately 15–25 km) from the tract centroid. Buffers were determined from access literature that focused on catchment zones and converted to times rather than distances (Widener, 2017; Yang et al., 2006; U.S. Department of Agriculture, 2022). While more comprehensive access models exist (Tenkanen et al., 2016), this straightforward and simpler approach is more accessible for practitioners to replicate and modify for their jurisdiction. Walking and driving buffers were created using *Network Analysis* in Arc Pro 3.1.

The proximity buffer was used for environmental capital variables to model what could affect a census tract, generally through environmental contamination. Following the calculation guidance of the EPA environmental justice screening tool (U.S. Environmental Protection Agency [EPA], 2022), buffer distances were applied uniformly to all census tracts for six variables. The urban–rural designation, access distances, and buffered variables were agreed upon with SCOR, and all variables that were buffered for TBRIC_A_ are indicated in Table S1.

### 4.3 Index construction and reliability analysis

In total, 90 variables were calculated for testing in CBRIC_A_, and 107 variables were calculated for testing in TBRIC_A_. Through the variable selection process with SCOR, 29 new variables were identified and tested in BRIC. After variables were calculated for each county and tract, the established BRIC methodology was used to calculate capital and total BRIC scores (Cutter et al., 2014). Raw values were normalized using min–max scaling to transform variables to a value between 0 (least resilient) and 1 (most resilient). Some variables were inverted, where a larger raw value would indicate less resilience (e.g., distance to the nearest hospital) by multiplying the raw value by negative one before normalization. In addition, any variables with a raw value variance less than 0.001 were removed from analysis.

All variables were then categorized by one of the six resilience capitals and tested against each other to determine their internal validity and similarity. If any two or more variables within a capital had a correlation coefficient above *r* = 0.7, all but one of the variables were removed or moved to a different capital. Lastly, Cronbach's alpha (*α*) statistic was used to measure the internal consistency of variables within each capital of CBRIC_A_ and TBRIC_A_ constructions. Cronbach's alpha values range from 0 to 1, with higher values demonstrating more internal consistency of the variables tested, meaning the variables assess the same concept. For this paper, alpha values greater than 0.70 are deemed acceptable, with values 0.60–0.69 questionable, 0.50–0.59 poor, and anything below 0.50 unacceptable (Cutter et al., 2014; Nardo et al., 2005). If a variable could conceptually be sorted into two or more capitals, it was tested in each to determine its best fit statistically.

An iterative process of reliability testing in SPSS, resorting, and removing variables lead to the final constructions of CBRIC_A_ and TBRIC_A_. Once variables were finalized, resilience capital scores were calculated by averaging the min–max standardized variables within each capital with a score range of 0 to 1. The total resilience score is the sum of all capital scores theoretically ranging from 0 to 6. No weighting is applied in the aggregation from variables to capital scores or from capital to BRIC scores.

### 4.4 Input data comparison to conventional BRIC

To understand how the actionable BRICs compare to previous iterations of BRIC, they are compared to the county BRIC used in the NRI (HVRI, 2024; Zuzak et al., 2023) and the recent downscale of BRIC published by Derakhshan et al. (2022). The conventional versions of BRIC (also referred to as CBRIC_C_ and TBRIC_C_) use contextual characteristics (e.g., age, disability, and proximity to county seat) and modifiable variables in their construction and are created for national application. Investigating the similarities and differences between the conventional and actionable constructions will show how much resilience measurements change depending on the inclusion of inherent characteristics, the use of national versus local datasets, and the selection of variables through their state‐level relevance.

Conventional BRICs are calculated according to variable lists and calculations described in their respective publications but with consistent and updated source data across CBRIC_C_ and TBRIC_C_. Table 1 explains where conventional and actionable BRICs differ in their source data or calculation due to suggested changes in the variable calculation process. Otherwise, CBRIC_C_ and TBRIC_C_ use the same source data as the actionable BRICs to highlight the effects of variable selection and calculation. A Cronbach's alpha and map were produced for each conventional index to compare with the actionable BRICs. If the conventional and actionable BRICs follow similar geography, the conventional BRICs could be considered suitable for measuring resilience in South Carolina. If they differ, South Carolina's place‐based resilience differs enough from national constructions of BRIC to warrant the customization. In addition, using statistical strength can justify utilizing one index over another.

**TABLE 1:** Variations in variable source and calculation between Conventional CBRIC and TBRIC and Actionable BRICs.

Variable	CBRIC_C_ Source *Calculation*	TBRIC_C_ Source *Calculation*	CBRIC_A_ and TBRIC_A_ Source *Calculation*
**Farmer's markets**	Census of Agriculture 2017 *Farms marketing products through commuity supported agriculture per capita*	USDA Local Food Directories Atlas 2020 *Farmers markets per capita*	SC DHEC 2022 and Certified SC 2022 *Farmers markets per capita*
**Mitigation funding**	FEMA 2010–2020 *Average amount ($) mitigation projects for a 10‐year period per capita*	SCEMD 2010–2020 *Average amount ($) mitigation projects for a 10‐year period per capita*	FEMA 2010–2020; SCOR *Average amount ($) mitigation projects for a 10‐year period per capit* *a* ^a^
			FEMA 2010–2020; SCOR *Average amount ($) mitigation planning projects for a 10‐year period per capita* ^a^
**Open space**	Not included	ESRI USA Parks *Percentage land in parks*	USGS Protected Areas Database 2022 *Percentage land in parks*
**Psychiatrist access**	County Health Ranking 2021 *Psychosocial support facilities per capita*	County Health Ranking 2021 *Psychosocial support facilities per capita*	SAMHSA 2021 *Psychosocial support facilities per capita*
**Schools**	National Center for Education Statistics, 2020–2021 *Number of schools per capita*	HIFLD 2021 *Numbers of public schools per capita*	South Carolina Schools Report Card, 2019–2020 *Students enrolled per 1000 school‐aged persons*

Abbreviations: DHEC, Department of Health and Environmental Control; FEMA, Federal Emergency Management Agency; HIFLD, Homeland Infrastructure Foundation‐Level Data; SAMHSA, Substance Abuse and Mental Health Services Administration; SCOR, South Carolina Office of Resilience; SCEMD, South Carolina Emergency Management Division; USDA, United States Department of Agriculture; USGS, United States Geological Survey.

^a^ This variable is split into two variables in the Actionable BRICs: 10‐year average per capita spending on mitigation projects and 10‐year average per capita spending specifically on mitigation planning projects.

## 5 RESULTS

Results from the actionable CBRIC and TBRIC construction are presented in comparison with the conventional CBRIC and TBRIC. The four indices are mapped and compared, followed by a deeper investigation of the geographic similarity between and the driving components of the actionable indices.

### 5.1 Actionable and conventional CBRIC and TBRIC constructions

The CBRIC_A_ construction consists of 48 variables, and TBRIC_A_ consists of 52 variables, with just over half shared with the conventional constructions (CBRIC = 28 and TBRIC = 29) (Table 2). Actionable TBRIC and CBRIC share 35 variables, and 10 of the variables in TBRIC_A_ are buffered using the new methodology accounting for tract geographies. All four BRIC constructions share 16 variables; however, five variables in actionable BRIC use local and/or updated data, reducing their direct overlap.

**TABLE 2:** Variables included in actionable CBRIC and actionable TBRIC constructions^a^.

Capital	Actionable CBRIC	Actionable TBRIC
**Social resilience**	Communication capacity	Communication capacity
	Health insurance	Educational attainment
	Life expectancy	Health insurance
	Physician access	High‐speed internet infrastructure
	Population stability	Housing affordability
	School capacity	Life expectancy
	TANF recipients	Physician access
		Supplemental Security Income
		TANF recipients
		Transportation access
**Economic resilience**	Building permits	Economic diversity
	Economic diversity	Employment rate
	Economic strength	Energy burden
	Economic strength II	Homeownership
	Employment rate	Low‐to‐moderate income
	Housing affordability	Non‐dependence on primary/tourism sectors
	Low‐to‐moderate income	
	Non‐dependence on primary/tourism sectors	
**Community capital**	Civic organizations	Civic organizations
	Community engagement	Community engagement
	Disaster volunteerism	Cultural heritage
	Food insecurity	Disaster volunteerism
	Political engagement	Large multi‐purpose retail (buffered)
	Sense of security	Religious organizations
	SNAP usage	Small business
	Walkability	
**Institutional resilience**	CRS discount	CRS discount
	Flood insurance coverage	Flood insurance coverage
	Institutional budget	Institutional budget
	Institutional budget II	Institutional budget II
	Jurisdictional uniformity	Jurisdictional uniformity
	Military employment	Military employment
	Mitigation planning spending	Mitigation planning spending
	Mitigation project spending	Mitigation project spending
	Registered voters	Population stability
		Registered voters
		SNAP usage
**Housing/infrastructural resilience**	Bridge rating	Dam hazard
	Evacuation routes	Food pantry access (buffered)
	Housing stock construction quality	Housing stock construction quality
	Internet access—connectivity	Local food suppliers (buffered)
	Medical care capacity	Medical care capacity
	Public transportation access	Public transportation access (buffered)
	Temporary housing availability	School capacity (buffered)
	Temporary shelter availability	Sturdier housing types
		Temporary housing availability
		Temporary shelter availability (buffered)
		Travel time to medical care
		Walkability
**Environmental resilience**	Air quality	Air quality
	Asthmatic population	Environmental contamination (buffered)
	Environmental contamination	Hazardous materials (buffered)
	Hazardous materials	Mining activity (buffered)
	Open space	Underground Storage Tanks
	Particulate matter	Water quality risk (buffered)
	Underground Storage Tanks	
	Water quality risk	

Abbreviations: CRS, Community Rating System; SNAP, Supplemental Nutritional Assistance Program; TANF, Temporary Assistance for Needy Families.

^a^ Conventional CBRIC and TBRIC variable lists can be found in their respective publications (Derakhshan et al., 2022; HVRI, 2024).

**TABLE 3:** Cronbach's alpha and number of variables (Vars) for actionable and conventional CBRICs and TBRICs by capital and total score.

Resilience capital	CBRIC_A_		TBRIC_A_		CBRIC_C_		TBRIC_C_	
	*α*	# Vars	*α*	# Vars	*α*	# Vars	*α*	# Vars
Social	0.747	7	0.839	10	0.767	10	0.746	9
Economic	0.708	8	0.672	6	0.532	10	0.322	12
Community capital	0.781	8	0.710	7	0.226	6	0.397	12
Institutional	0.696	9	0.643	11	−0.614	10	0.217	10
Housing/infrastructural	0.702	8	0.740	12	0.614	9	0.639	13
Environmental	0.754	8	0.700	6	0.422	5	0.479	9
All Variables	0.928	48	0.829	52	0.655	50	0.576	65

Due to the prioritization of the 0.7 Cronbach's alpha threshold for actionable BRIC capitals, there are marked differences in some capital constructions when compared to conventional BRICs. Economic capital in the conventional BRIC contains variables on income equality and business size. The actionable BRICs shift to economic diversity, GDP, and housing burden variables. Community capital shifts slightly to include variables historically included in institutional and economic capitals but also applicable to measuring community capital theoretically (e.g., political engagement and small business). Lastly, both conventional BRIC's environmental capitals include some environmental pollution metrics and variables measuring pervious surfaces and energy and water use, which fall out of the actionable constructions. CBRIC_A_ and TBRIC_A_ environmental capitals are more similar to measures of environmental pollution and environmental justice than to previous iterations of BRIC, primarily due to the lack of adequate statewide measures of environmental resilience.

The CBRIC_A_ total scores range from 1.50 to 4.50, the largest range of any of the constructions, with an average total resilience score of 2.68. All but one resilience capital, institutional resilience, has a Cronbach's alpha of *α* < 0.70, and the total index has an alpha of *α* = 0.928 (Table 3). Compared to CBRIC_C_ (range 2.04–3.53; *x̄* = 2.72), CBRIC_A_ has a higher range and performs better statistically in this study area. TBRIC_A_ total scores range from 2.43 to 4.06, and an average total resilience score of 3.08. While two capitals (economic and institutional) do not meet the *α* > 0.70 threshold, the overall TBRIC_A_ has an alpha *α* = 0.829. TBRIC_C_ (2.46–3.54; *x̄* = 2.87) performs poorly statistically, apart from the social capital (*α* = 0.746), and has the lowest total index Cronbach's alpha for the entire index (*α* = 0.576) out of the four indices computed.

Reliability results are consistent across all four indices for social and housing/infrastructure capitals, but actionable BRICs improve upon community and environmental capital. Institutional resilience does not meet the threshold of 0.7 in any of the BRIC constructions but by far performs the worst in the CBRIC_C_ construction with a negative alpha value (*α* = −0.614), which is largely due to correlations between mitigation funding, jurisdictional uniformity, and population stability for this region. While mitigation funding and jurisdictional uniformity are also included in the custom BRICs, mitigation funding is split into two variables (projects and planning), which removes the strong correlation, improving the Cronbach's Alpha. Overall, the reliability testing shows that some capitals from national, conventional constructions represent specific capitals of resilience well in South Carolina, but the actionable indices perform better overall and improve upon their conventional counterparts.

#### 5.1.1 Correlations between BRICs

To compare the two actionable constructions and the two conventional constructions using correlation, sample sizes must be equal. TBRIC scores are averaged by county to compare all four constructions for county geography. Then, CBRIC scores are allocated to all tracts within the county to compare all four constructions at the tract scale. According to the MAUP, as aggregation increases, we expect to see correlations increase as well. A Pearson's correlation shows that when CBRICs and aggregated TBRICs are compared, they all have a significant (*p* < 0.01) and positive relationship (*r* ≥ 0.68) (Table 4). The two actionable indices have the strongest relationship (*r* = 0.963), which is much stronger than the correlation between the two conventional constructions (*r* = 0.680). This is partially due to the actionable constructions drawing on the same larger set of variables, whereas TBRIC_C_ is based on CBRIC_C_ but includes new variables specific to the tract construction.

**TABLE 4:** Pearson's Correlations when TBRIC is aggregated to county geography and when CBRIC is disaggregated to tract geography.

County geography (aggregation)				
	CBRIC_A_	TBRIC_A_	CBRIC_C_	TBRIC_C_
**CBRIC_A_**	–			
**TBRIC_A_**	0.963^**^	–		
**CBRIC_C_**	0.766^**^	0.700^**^	–	
**TBRIC_C_**	0.763^**^	0.680^**^	0.693^**^	–

Tract geography (downscale)				
	CBRIC_A_	TBRIC_A_	CBRIC_C_	TBRIC_C_
**CBRIC_A_**	–			
**TBRIC_A_**	0.629^**^	–		
**CBRIC_C_**	0.773^**^	0.434^**^	–	
**TBRIC_C_**	0.577^**^	0.829^**^	0.523^**^	–

** *p* < 0.01.

At the tract level, when TBRICs are correlated to downscaled CBRICs, all relationships are, again, positive and significant (*p* < 0.01), but to a weaker degree, which is expected due to CBRIC's inability to measure subcounty variability. When county scores are disaggregated to tract, CBRIC_A_ and TBRIC_A_’s relationship weakens slightly (*r* = 0.629), but conventional and actionable TBRIC have a strong positive relationship (*r* = 0.829). The two conventional constructions have only a moderate relationship at the tract level (*r* = 0.523), indicating that their scores are not as similar as the two actionable indices.

As expected, county geography has generally higher correlation coefficients than tract geography. CBRIC_A_ and CBRIC_C_ are very similar, and TBRIC_A_ and TBRIC_C_ are very similar, meaning that the actionable indices are not very different from their conventional counterparts. However, the actionable BRICs have higher correlations across scales (i.e., when aggregated and downscaled) than the conventional BRICs, meaning the actionable BRICs are more like each other even when subject to scale effects.

#### 5.1.2 Visualizing BRICs

**FIGURE 3:**
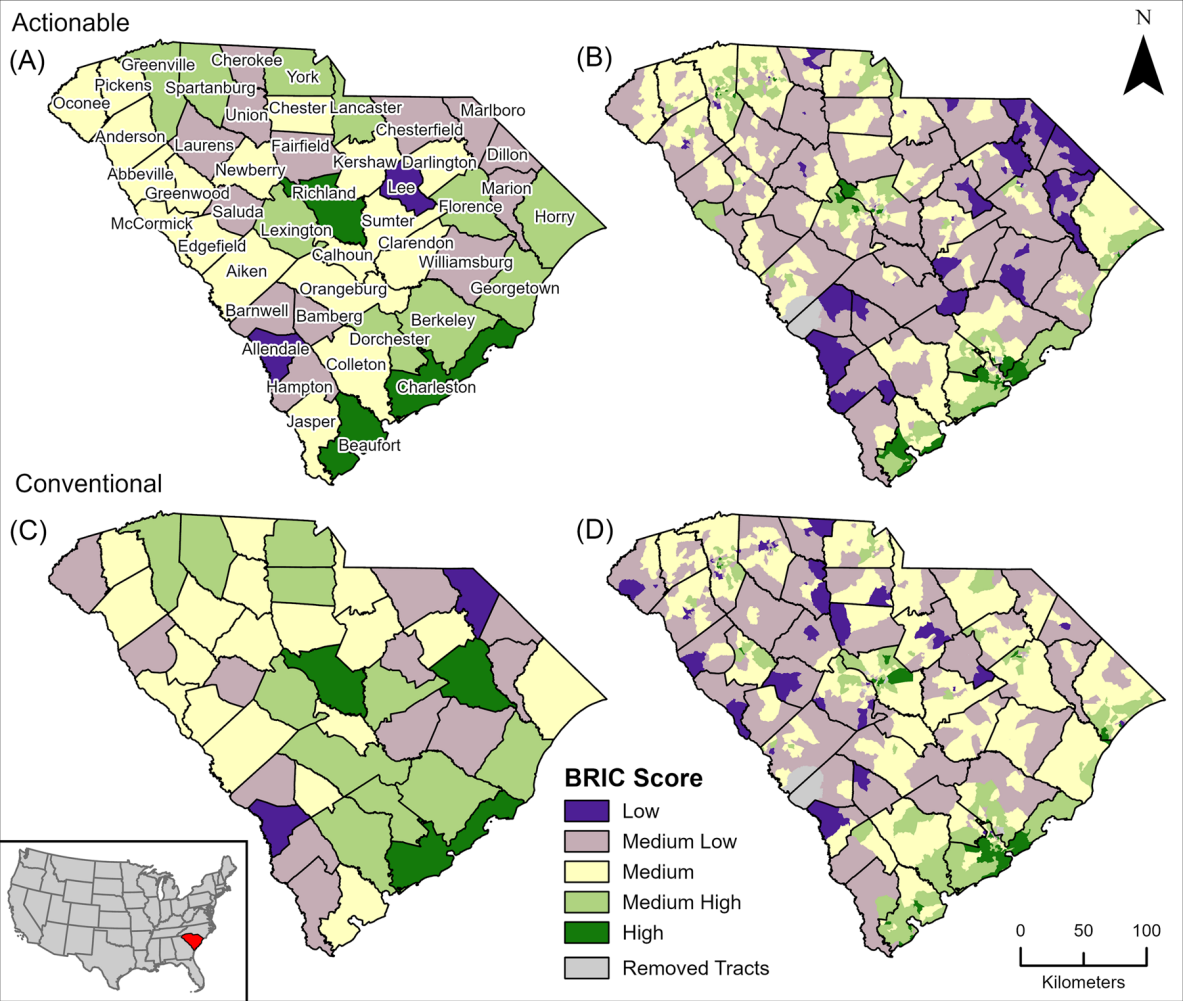
Actionable (A) CBRIC and (B) TBRIC and conventional (C) CBRIC and (D) TBRIC mapped by standard deviations.

Since BRIC is a metric measuring differences between places, correlations do not fully explain the similarities and differences between actionable and conventional BRICs. CBRIC and TBRIC scores are mapped by the standard deviation to preserve their underlying distribution (Figure 3). Medium‐resilience areas have a BRIC score between −0.5 and 0.5 standard deviations from the mean. Medium‐low and medium‐high resilience areas have a BRIC score between ±0.5 and ±1.5 standard deviations from the mean, and the areas with the highest and lowest resilience have scores further than ±1.5 standard deviations from the mean.

Echoing previous research (Cutter et al., 2016), there is an urban–rural resilience divide observed in South Carolina. Urban counties, including Charleston County, which contains the state's largest city, and Richland County, where the state's capital is located, have both majority high‐ and medium‐high resilience in all constructions. Allendale County has the lowest resilience across both county constructions and Lee and Marlboro Counties, also rural counties, are of the lowest resilience in CBRIC_A_ and CBRIC_C_, respectively. In general, rural areas have lower resilience due to limited access to services and resources, differences in institutional assistance, and population and employment characteristics (Cutter et al., 2016; Seong et al., 2022). Many counties shift one category higher or lower between the two county constructions (e.g., Florence County has medium‐high resilience in CBRIC_A_ and high resilience in CBRIC_C_). Regionally, the counties between Richland and Charleston Counties are closer to medium resilience in CBRIC_A_ and higher or lower resilience in CBRIC_C_. The county with the largest change in its resilience category is Beaufort, which has high resilience in CBRIC_A_ and medium resilience in the CBRIC_C_. This is due to increased community and institutional capital scores for the county in CBRIC_A_.

Though TBRICs contain some downscaled county variables (e.g., County GDP), there are enough tract and sub‐tract scale variables included in TBRIC that subcounty variability is clear. Considering the two TBRICs are statistically similar, their spatial patterns of resilience are very different, especially in geographies of low and medium‐low resilience. While urban areas have higher resilience in both tract constructions, TBRIC_A_ has lower resilience in coastal and just inland regions, crossing the state from Dillon County on the North Carolina border to Allendale County on the Georgia border. This is a different geography of low resilience compared to TBRIC_C_, where regions of lower resilience are concentrated in the northwestern counties and along the South Carolina‐Georgia border.

**FIGURE 4:**
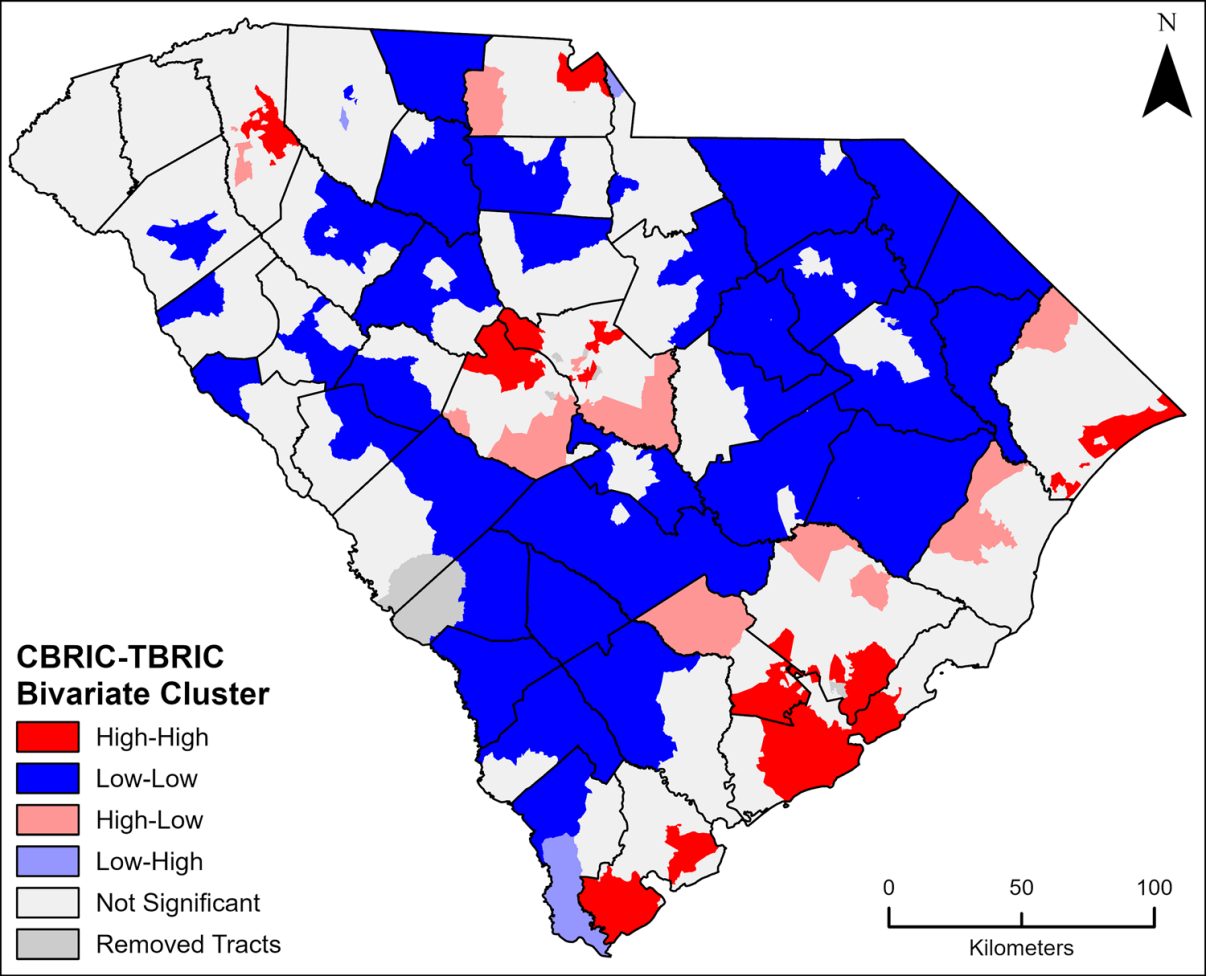
Bivariate local Moran's *I* showing common clusters of high and low values between disaggregated CBRIC_A_ and TBRIC_A_.

To further assess the spatial similarity between CBRIC_A_ and TBRIC_A_, bivariate local Moran's *I* (Queen Contiguity weighting) was used in GeoDa 1.20 to compare clusters of high and low actionable BRIC scores across the two scales of analysis (Figure 4). To compare the two, only tract geography is used, using county scores assigned to all tracts within the county compared to TBRIC_A_, preserving the subcounty variability in TBRIC_A_. Most tracts (58.2%) do not show a significant association, but there are regions of high‐high clusters in urban and suburban areas and low‐low clusters in rural areas. These common high and low clusters account for 38.2% of tracts, whereas there are very few outliers.

Common clusters of high BRIC scores are concentrated along the coast and in urban areas, and low clusters follow the shift from northeast to southwest in low rural resilience between Dillon and Allendale Counties. Outliers where CBRIC_A_ is higher than TBRIC_A_ are generally in the rural tracts of higher scoring counties due to the aggregation in CBRIC_A_, masking subcounty variability. An example of this is Berkeley County, where in CBRIC_A_, the entire county has medium‐low resilience, but in TBRIC_A_, tracts in the southern part of the county (closer to the City of Charleston) have high BRIC scores (high CBRIC_A_‐high TBRIC_A_ cluster) and as you move away from the city to more rural areas in the northern part of the county, tracts have medium and medium‐low resilience (high CBRIC_A_‐low TBRIC_A_ cluster).

Though the bivariate Moran's *I* highlight some areas where CBRIC_A_ and TBRIC_A_ differ, the correlation analysis and visualization results show a larger level of agreement than previous investigations of scale in other regions and countries. For example, the initial downscale of BRIC in the Gulf Coast found a strong relationship between CBRIC and an aggregated TBRIC (Spearman's rho = 0.796, *p* < 0.01), but that relationship dissipated when CBRIC was assigned to tracts (Spearman's rho = 0.483, *p* < 0.01) (Derakhshan et al., 2022). In addition, Chu et al. (2021) discuss scale effects at length in their social resilience index applied in Vancouver, Canada, and find significant disparities in the geography of resilience measured at different scales. This is not the case for South Carolina.

### 5.2 Components of actionable CBRIC and TBRIC

Drivers of resilience depend on scale as well as place. While CBRIC_A_ and TBRIC_A_ are derived from the same larger set of variables and are similar statistically and spatially, their final constructions are different. To understand how the two scales of actionable BRIC identify different drivers, descriptive statistics and Pearson's correlations between capital scores and the total BRIC score are presented for all counties in CBRIC and all tracts in TBRIC (Table 5).

**TABLE 5:** Descriptive statistics and Pearson's Correlation coefficients for CBRIC_A_ and TBRIC_A_ capitals.

Actionable CBRIC						
	Social	Economic	Community	Institutional	Housing/infrastructural	Environmental
Minimum	0.202	0.183	0.135	0.141	0.117	0.225
Maximum	0.741	0.748	0.865	0.680	0.717	0.853
Mean	0.506	0.508	0.458	0.308	0.333	0.566
Standard deviation	0.020	0.019	0.021	0.018	0.019	0.021
Correlation coefficient^a^	0.846	0.794	0.892	0.714	0.778	0.844

Actionable TBRIC						
	Social	Economic	Community	Institutional	Housing/infrastructural	Environmental
Minimum	0.354	0.403	0.001	0.136	0.166	0.373
Maximum	0.859	0.959	0.803	0.598	0.699	1.000
Mean	0.650	0.792	0.047	0.319	0.320	0.956
Standard deviation	0.0028	0.0023	0.0015	0.0022	0.0020	0.0014
Correlation coefficient^b^	0.882	0.577	0.225	0.679	0.418	0.166

^a^ When correlated with the Total CBRIC_A_ score.

^b^ When correlated with the Total TBRIC_A_ score.

Due to TBRIC_A_ having a smaller geography and less aggregation, thus preserving more variability, capitals have higher ranges than in CBRIC_A_. Mean capital scores and standard deviations show the capitals in CBRIC_A_ and TBRIC_A_ with outliers in their component variables, causing the rest of the min–max values to cluster near the other end of the range. For example, TBRIC_A_’s environmental resilience has a very high mean score (*x̄* = 0.956) and a low standard deviation (*σ* = 0.0014), meaning that most tracts have a very high environmental capital score, even though tract scores range from 0.373 to 1. In CBRIC_A_, environmental resilience also has the highest mean score of all capitals (*x̄* = 0.566), but it's much closer to 0.5 (the middle of the theoretical capital range), and the standard deviation is higher (*σ* = 0.021), indicating a larger spread of values. Having outliers of low environmental resilience in TBRIC_A_ is an easy way to identify the priorities for environmental resilience projects, as few tracts have very low environmental resilience. However, since BRIC is a relative metric if the tracts with low environmental resilience are improved (i.e., raise their score), the environmental capital could change drastically for the entire state. The opposite is true for community capital in TBRIC, which has, on average, very low scores, with high‐scoring outliers that can be examples of how to improve community capital statewide. However, improvement in one tract may not trigger drastic changes in the capital overall.

The correlation between each scale's capital score and the total BRIC score can help explain what capitals and variables most influence the state's resilience scores. Of the six capitals in CBRIC_A_, community, social, and environmental capital have the highest correlation coefficients with the total CBRIC score, though all capitals have a correlation coefficient above 0.7. At the tract scale, social and institutional resilience have the highest correlations with the total TBRIC score, and community and environmental resilience have the lowest. While the two actionable indices are very similar, the drivers of resilience differ depending on the aggregation scale.

## 6 DISCUSSION AND CONCLUSIONS

This paper asks how a state‐level resilience index can be adapted to measure relevant and modifiable community characteristics for use by a state resilience office. We addressed this question by constructing two actionable BRICs that prioritize locally relevant characteristics and utilize variables that can be changed through nonlegislative action at the state or local level. The result is an actionable metric co‐developed with SCOR to monitor modifiable community resilience in South Carolina. This new metric takes a practical perspective, prioritizing interventions and tracking resilience from SCOR's point of view to better track success and highlight potential areas of most need.

Methodologically, the approach to creating these actionable BRICs differs from conventional approaches in three key ways. First, actionable BRICs were co‐created with SCOR to prioritize local datasets, include modifiable variables, and identify relevant variables for South Carolina. Second, selected tract‐level variables were calculated with a proximity or access buffer that addressed the non‐jurisdictional boundaries of census tracts. Third, the number of variables retained in actionable BRICs was determined through correlation analysis and Cronbach's alpha testing, with variables removed and resorted until the capital reached or nearly reached the 0.7 threshold. The conventional BRIC constructions did remove some variables during analysis, but far fewer, prioritizing the total index's Cronbach's alpha result and retaining variables rather than maximizing the internal consistency of each capital independently.

Our second research question asks how this resulting actionable index compares to conventionally used resilience indices. The comparisons between actionable and conventional BRICs highlight their different approaches and uses in scales of community resilience measurement. In actionable BRIC, the geography of medium‐low and lowest resilience shifts to the state's southeastern region, indicating that integrating place‐based measures of resilience highlights different areas of concern in the state than nationally focused constructions. CBRIC_A_ and TBRIC_A_ have stronger correlations when aggregated to county and downscaled to tract, and they highlight similar areas of high and low resilience, showing limited scale effects compared to conventional BRICs, especially in areas of highest and lowest resilience. Lastly, the differences in spatial patterns of resilience between conventional and actionable BRICs highlight a difference in national versus place‐based perspectives for resilience measures and the mixture of actionable and contextual variables versus purely actionable measures of resilience.

The methodological changes from conventional to actionable constructions have their strengths and weaknesses. Coproducing the variable list with a relevant agency like SCOR is a replicable method for creating a usable and relevant resilience index that can be integrated into state‐level resilience planning. We completed the calculation and analysis components of the index construction but prioritized data and calculation methods that were as straightforward as possible to encourage replication within agencies rather than requiring outside experts. For example, access from tracts can be measured in many ways, with our method selecting the most approachable to non‐experts.

While the actionable indices were similar to the conventional metric that uses both contextual and actionable variables, more sensitivity testing and analysis should investigate the effect of removing these contextual factors of resilience and adding buffers to tract geography. Through this methodology, many variables were removed from the actionable BRIC constructions that are still relevant to resilience in the state, but that did not fit into the index statistically. This is reflected in the shift of the environmental resilience capital, which exemplifies the challenges of capturing absorptive capacity and other dimensions of environmental resilience for an entire state (also true for a national approach). In this case, environmental resilience and any removed variables could be better suited to a bottom‐up, participatory approach. Such an approach can also capture local drivers of resilience that were not relevant for the entire state of South Carolina for this top‐down measurement. Integrating top‐down and bottom‐up resilience perspectives and measurement is needed to further understand the role of scale, actionable measurement, and the applicability of different resilience variables at various scales of analysis. Ignoring local drivers of resilience and relying solely on top‐down indices can lead to the misallocation of funds, and a top‐down approach to identifying resilience projects can further vulnerabilities, keeping some individuals from becoming more resilient (Ranganathan & Bratman, 2021).

Actionable CBRIC and TBRIC utilize variables that can be affected by direct actions at the state and local levels. Follow‐up assessments on how the indices are implemented and their usefulness in state‐level planning and decision‐making will validate or lead to further adjustments of BRIC to align and improve the usability of these index tools (Wall et al., 2017). Scale and zoning effects will always exist in indices such as BRIC, which rely on aggregated data for their construction. The tract scale is the smallest unit of analysis to compute BRIC without major data availability and accuracy issues, suggesting that it is the most accurate assessment of resilience (Schuurman et al., 2007). However, tracts do not align with jurisdictional boundaries, and though CBRIC loses the subcounty variability that TBRIC provides, CBRIC is easier and faster to compute, requiring less geospatial data analysis and having more stable boundaries over time. Additional research should explore the potential for using other jurisdictional boundaries, such as incorporated areas, to represent where municipalities have agency rather than the state and county perspective.

In addition, tract‐level indices require further research and suitability studies to determine when and how they can be designed to reflect resilience across a large area, such as a state or nation, including whether their benefits outweigh the time and resources required to calculate them. BRIC is only one of many approaches to resilience measurement. In the future, it would be helpful to compare the custom BRICs to other resilience metrics and external proxies of resilience and recovery for South Carolina further to investigate the relevancy and validity of this metric.

The need for state and local agencies to measure and understand their community's resilience is still developing. Many places are writing their first resilience plans and developing initial metrics to track their success (Malecha et al., 2024). Here, we provide a methodology that adapts a research‐developed community resilience metric, BRIC, to the practical needs of SCOR and has implications for state and sub‐state applications. Customizing CBRIC and TBRIC to integrate actionable variables that reflect SCOR's understanding of community resilience in South Carolina creates an opportunity for state‐level resilience tracking and planning. These actionable indices statistically improve upon their conventional counterparts and create usable tools to understand and communicate resilience priorities as a whole or broken down by capital to develop specific enhancement strategies over time. The variables chosen for testing reflect SCOR's understanding of community resilience in South Carolina rather than a national perspective. As more places look to already developed resilience metrics to suit their needs, tailoring an established metric to a state‐specific and action‐oriented perspective is replicable for various study areas and scales.

## CONFLICT OF INTEREST STATEMENT

The authors declare no conflicts of interest.

## Supporting information



Supplementary Material

## Data Availability

Capital and Total BRIC scores for all actionable and conventional constructions are available at https://doi.org/10.5281/zenodo.11094746.

## Footnotes

1 Since 2020, SCOR was working on the state's first‐ever resilience plan and, in the process, contracted HVRI to create a custom BRIC index for South Carolina. SCOR took part in developing this approach by assisting in variable identification and data acquisition specifically for South Carolina and by serving as an advisor on the practicalities of the measurement approach for resilience planning.

2 A census‐designated place (CDP) is a geographic area defined by the U.S. Census Bureau for statistical purposes. Unlike incorporated cities, CDPs lack their own local government but are used to gather data about unincorporated communities.
