# Supplementary material for: A community resilience index for place‐based actionable metrics
Source: Risk Anal. 2024 Dec 6;45(7):1648–61. doi: 10.1111/risa.17684 (PMC12396936; doi:10.1111/risa.17684)
Supplement: Supplementary file 1 — Supplementary Material [file RISA-45-1648-s001.pdf]

Supplementary Material

Table S1. All variables tested in actionable County (CBRIC) and Tract (TBRIC) BRIC. New variables are italicized.

Variable	Calculation	CBRIC/TBRIC Source
Air quality†	# Air polluting facilities per 1,000 persons (Inverted)	EPA FRS Geodatabase 2023
Asthmatic population	*% Population with asthma (Inverted)*	*PLACES 2022*
Bridge rating	Average sufficiency rating of all bridges	National Bridge Inventory 2018
Building permits	Five-year average number of building permits per 1,000 persons	Building Permits Survey 2016-20
Civic organizations*	# Civic organizations per 1,000 persons	ESRI Business Analyst 2021
Communication capacity	% Households with telephone service available	ACS 5Y 2020/2019
Community engagement	# Art, entertainment, and recreation establishments per 1,000 persons	ESRI Business Analyst 2021
Community housing & relief services	# Community housing and emergency & relief services per 1,000 persons	ESRI Business Analyst 2021
Crop insurance	# Crop insurance policies per square mile [CBRIC Only]	Environmental Working Group Farm Subsidies 2014
CRS discount	*Average CRS Discount Rate*	*OpenFEMA 2023*
Cultural heritage	# Museums, historical sites, and similar institutions per 1,000 persons	ESRI Business Analyst 2021
Dam age	Average age of dams (Inverted)	National Inventory of Dams 2020
Dam hazard	*# Dams that are designated high hazard or unsatisfactory or unknown condition divided by average number of dams (Inverted)*	*National Inventory of Dams 2020*
Disaster aid experience	# Presidential Disaster Declarations divided by # of loss-causing hazard events for ten-year period	FEMA PDD database; SHELDUS 2010-2020
Disaster volunteerism	# AmeriCorps volunteers per 1,000 persons	CNCS 2021
Economic diversity	*Shannon-Wiener Diversity Index of employment across sectors*	*ACS 5Y 2020/2019*
Economic strength I	*Gross Domestic Product (GDP) per capita*	*BEA 2021*
Economic strength II	*% Change in GDP from 2018-2021*	*BEA 2021*
Educational attainment	% Population with at least some college education	ACS 5Y 2020/2019
Efficient energy use	Energy Use per Utility Customer (Inverted)	City & County Energy Profiles 2021
Emergency services (police/fire)	# Emergency services per 1000 persons	HIFLD 2021
Employment rate	% Labor force employed	ACS 5Y 2020/2019
Energy burden	Average Energy Burden (% income) (Inverted)	Low-Income Energy Affordability Data 2018
English language competency	% Population that are proficient English speakers	ACS 5Y 2020/2019
Environmental contamination†	# Superfund or LQG hazardous waste facilities per 1,000 persons (Inverted)	EPA FRS Geodatabase 2023
Evacuation routes	Intersection density (# real nodes/tract area in sq. miles)	National Neighborhood Data Archive 2021
Flood insurance coverage	% Housing units covered by National Flood Insurance Program	FEMA NFIP 2021
Food insecurity	% Population food insecure (Inverted)	Feeding America 2021
*Food pantry access**	*# Food pantries per 1,000 persons*	*DHEC 2022*
Gender income equality	% Absolute difference between male and female median income divided by annual income (Inverted)	ACS 5Y 2020/2019
Government employees	*% Labor force working for the government*	*ACS 5Y 2020/2019*
Hazardous materials†	*# Hazardous material spills in last 10 years per 1,000 persons (Inverted)*	*DOT 2010-2020*
Health insurance	% Population under age 65 with health insurance	ACS 5Y 2020/2019
High-speed internet infrastructure	% Households with broadband internet subscription	ACS 5Y 2020/2019
Homeownership	% Owner-occupied housing units	ACS 5Y 2020/2019
Hospital access*	# Hospitals per 1,000 persons	HIFLD 2021
Housing affordability	*% Housing units spending less than 30% of income on mortgage or rent*	*ACS 5Y 2020/2019*
Housing stock construction quality	% Housing units built prior to 1970 or after 2000	ACS 5Y 2020/2019

Income-to-loan ratio	Average income to mortgage loan ratio	ACS 5Y 2020/2019
Institutional budget I	*County revenue per capita*	*South Carolina Association of Counties 2022*
Institutional budget II	*County revenue divided by county expenditures*	*South Carolina Association of Counties 2022*
Internet access - connectivity	# Actual connections per 1,000 households	National Neighborhood Data Archive (NaNDA) 2014-2018
Jurisdictional uniformity	# Governments and special districts per 10,000 persons (Inverted)	Census of Governments 2012 and 2017
Land in flood zone	Percent land in a 1% 2022 Flood Event (Inverted)	SCOR 2023
Large multi-purpose retail*	# Large retail stores per 1,000 persons	ESRI Business Analyst 2021
Life Expectancy	*Life expectancy*	*County Health Rankings 2016-2018/CDC NCHS 2015*
Local food suppliers*	# Farmers' markets per 1,000 persons	DHEC 2022 & Certified SC 2022
Low-to-Moderate Income	*% Households designated as Low-to-Moderate (LMI) income by HUD (Inverted)*	*HUD 2015*
Medical care capacity	# Hospital beds per 1,000 persons	HIFLD 2021
Medical device dependence	*% Population dependent on medical support devices*	*HHS 2022*
Military employment	% Employed armed forces	ACS 5Y 2020/2019
Mining activity†	*# Mines per 1,000 persons (Inverted)*	*SCDHEC 2023*
Mitigation planning Spending	*Ten-year average per capita spending for mitigation planning projects*	*FEMA HMGP 2011-2020 & SCOR*
Mitigation project spending	Ten-year average per capita spending for mitigation projects	FEMA HMGP 2011-2020 & SCOR
Natural flood buffers	2019 wetland area/2008 wetland area	National Land Cover Dataset 2020
Non-dependence on primary/tourism sectors	% Employees not in farming, fishing, forestry, extractive industry, or tourism	ACS 5Y 2020/2019
Nuclear accident planning	% Population within 10 miles of nuclear plant	HIFLD Nuclear Power Plants Database 2020 & Census 2020
Open space	% Land in parks	USGS Protected Areas Database 2022
Particulate matter	*Average particulate matter value (Inverted)*	*EJSCREEN 2022*
Pervious surface change	Change in perviousness	National Land Cover Dataset 2008-2019
Pervious surfaces	Average percent pervious surfaces	National Land Cover Dataset 2020
*Pharmacy access**	*# Pharmacies within geography per 1,000 persons*	*ESRI Business Analyst 2021*
Physician access	# Physicians per 1,000 persons	ACS 5Y 2020/2019
Place security/Evictions	# Evictions per 1000 persons (Inverted)	Princeton Eviction Lab 2016
Political Engagement	% Voter participation in a Presidential election	SCVOTES 2020
Population stability	Population change over previous five-year period (Inverted)	ACS 5Y 2014 and ACS 5Y 2019
Psychologist access*	# Psychosocial support facilities per 10,000 persons	SAMHSA 2021
*Public transportation access**	*# Transit stops per 1,000 persons*	*Bureau of Transportation Statistics 2022*
Race/ethnicity income equality	Gini coefficient (Inverted)	ACS 5Y 2020/2019
Registered voters	*% Population over 18 registered to vote*	*SC Votes 2020 election*
Religious organizations*	# Religious organizations per 1,000 persons	ESRI Business Analyst 2021
Sales rate	Average sales volume divided by number of businesses	ESRI Business Analyst 2021
*School capacity**	*# Students enrolled per 1,000 school-aged persons (18 and under)*	*SC Schools Report Card, 2019-20 & ACS 5Y 2020/2019*
Sense of security	Crime rate (property and violent crime), 5-year average, per 1,000 persons (Inverted)	FBI 2015-2019
Small business	# Small business establishments (10 or less employees) per 1,000 persons	ESRI Business Analyst 2021
SNAP usage	*% Households receiving SNAP benefits (Inverted)*	*ACS 5Y 2020/2019*

Social assistance services	# Social assistance services per 1,000 persons	ESRI Business Analyst 2021
Structures in Flood Zone	*% Structures at risk of inundation in a 1% 2022 Flood Event*	*SCOR 2023*
Sturdier housing types	% Housing units not mobile homes	ACS 5Y 2020/2019
Supplemental Security Income	*% Households receiving SSI benefits (Inverted)*	*ACS 5Y 2020/2019*
TANF Recipients	*% Population receiving TANF benefits (Inverted)*	*SC DSS 2019-20*
Temporary housing availability	# Vacant rental units per 1,000 persons	ACS 5Y 2020/2019
Temporary shelter availability*	# Hotels/motels per 1,000 persons	ESRI Business Analyst 2021
Trade Employment	*% Population employed as specialty trade contractors*	*BLS 2021*
Transportation access	% Households with at least one vehicle	ACS 5Y 2020/2019
Travel time to medical care	Average travel time from tract centroid (driving minutes) to nearest hospital (Inverted) [TBRIC Only]	HIFLD 2021
Underground Storage Tanks†	*# Open underground storage tanks per 1,000 persons (Inverted)*	*EPA UST Finder 2023*
Urban flooding	Average Flash Flood Potential Index value (Inverted)	SCEMD HIRA 2023, HVRI
Walkability	*Average walkability score*	*EPA 2019*
Water quality risk†	# NPDES point source pollution facilities within geography per 1,000 persons (Inverted)	EPA FRS Geodatabase 2023
Water Stress	Water Supply Stress Index	Water Risk Atlas 2019

* variable was tested in TBRIC using a 15-minute walk (urban) or 30-minute drive (rural) access buffer.
† variable was tested in TBRIC using a uniform proximity buffer on the census tract.

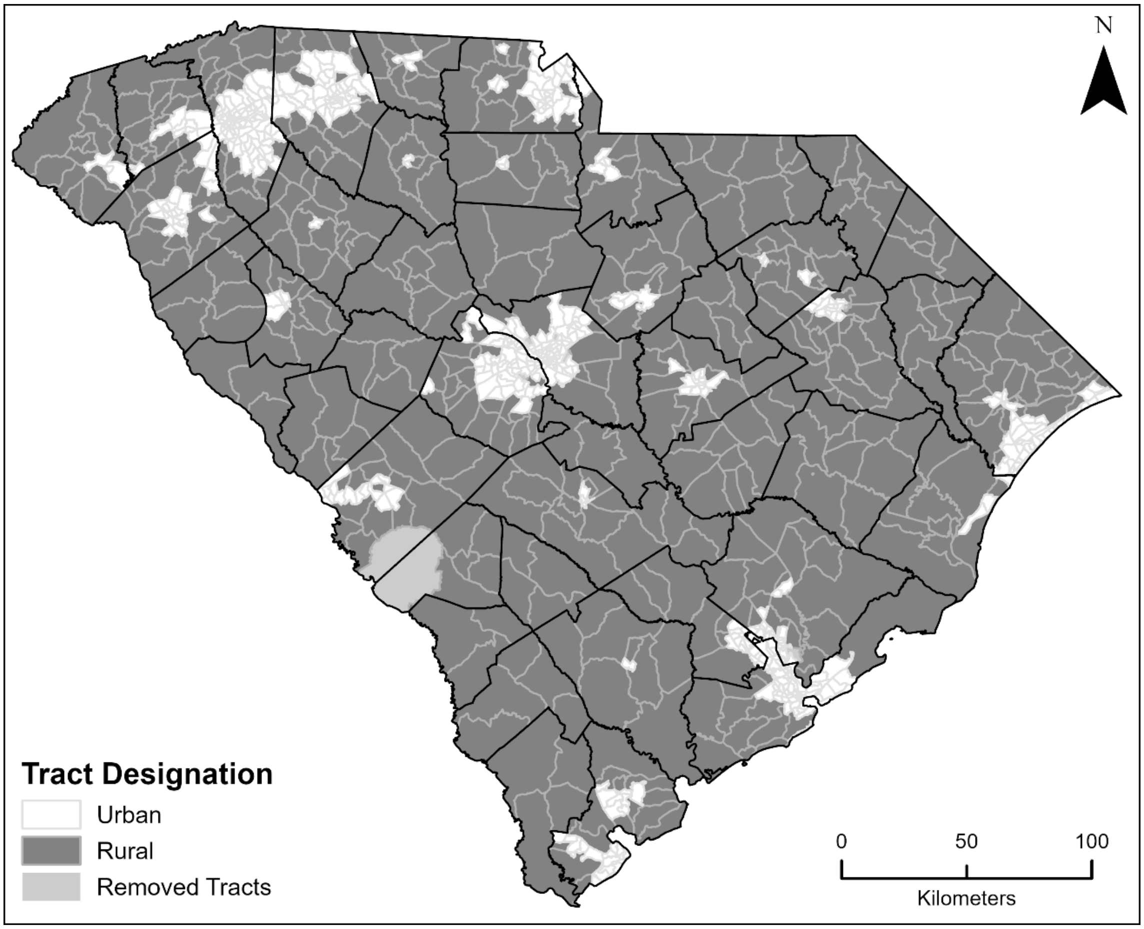

Figure S1. South Carolina census tracts designated as urban or rural for this study. Tracts removed from analysis have zero population or zero housing units.

Capital and Total BRIC scores for all actionable and conventional constructions are available at http://doi.org/10.5281/zenodo.11094746.
